# Prevalence of Behavioral Addictions and Their Relationship With Stress and Anxiety Among Medical Students in Saudi Arabia: A Cross-Sectional Study

**DOI:** 10.3389/fpsyt.2021.727798

**Published:** 2021-08-17

**Authors:** Alqassem Y. Hakami, Rami Ghazi Ahmad, Abdullah Alsharif, Alaa Ashqar, Fahad A. AlHarbi, Mohammed Sayes, Anas Bafail, Ali Alqrni, Mohammed A. Khan

**Affiliations:** ^1^College of Medicine, King Saud bin Abdulaziz University for Health Sciences, Jeddah, Saudi Arabia; ^2^King Abdullah International Medical Research Center, Jeddah, Saudi Arabia; ^3^Psychiatry Section, Department of Medicine, Ministry of National Guards-Health Affairs, Jeddah, Saudi Arabia

**Keywords:** behavioral addictions, internet, gaming, addiction disorder, stress, anxiety, medical students

## Abstract

Behavioral addiction is identified as any compulsive, repeated, and persistent behavior that leads to significant and functionally impairing harm or distress. The aim of this study is to determine the prevalence of internet, video-gaming, and pornography addictions among medical students in Western region. In addition, we intend to investigate the relationship between these behavioral addictions with stress and anxiety. Our study was a cross-sectional study with a sample size of 225. The study participants were medical students in their 3rd, 4th, and 5th academic years from five different medical colleges in Western region. The questionnaire included demographics and adapted five different pre-validated scales: Young's Internet Addiction Test – Short Version (IAT-SV), Internet Gaming Disorder Scale 9 – Short Form (IGDS9-SF), (PPC scale), Perceived Stress Scale (PSS), and Generalized Anxiety Disorder 7-item scale (GAD-7). The IAT-SV scale showed: 71 (31.6%) of the participants had normal internet usage, 51 (22.7%) participants showed problematic usage, and 103 (45.8%) used the internet pathologically. The IGDS9-SF scale had observed the following values: 220 participants (97.8%) were non-disordered, and 5 participants (2.2%) were found to be disordered. Statistical analysis showed a highly significant association between stress and problematic pornography consumption (*P* < 0.01), and internet addiction (*P* <0.001). Moreover, there was a significant association between anxiety and internet gaming disorder (*P* < 0.01). This study showed high prevalence of internet addiction and low prevalence of internet gaming disorder. Also, it gave more understanding to a possible association between these behavioral addictions with stress and anxiety.

## Introduction

“Addiction is a treatable, chronic medical disease involving complex interactions among brain circuits, genetics, the environment, and an individual's life experiences” (1). Recently, non-substance or behavioral addictions have been increasingly acknowledged and studied; however, epidemiological and neurobiological data are still not fully established. Recently, a group of researchers suggested a more specific definition for behavioral addiction as follows: “Behavioral addiction is known as any compulsive, repeated, and persistent behavior that leads to significant and functionally impairing harm or distress” (2). In addition, to consider any behavior as an addiction, it must not be explained by an underlying illness, done willingly or done as a coping method, and it must cause significant functional impairment or distress (2).

Behavioral addictions (e.g., gambling disorder, internet addiction, video-gaming addiction) are so-called because they exhibit many addictive qualities. However, this is not conclusive to the addictive nature of these behaviors but is rather indicative of problematic use. First, behavioral addictions are irresistible and impulsive in nature, which ultimately result in functional impairment (3). Second, most behavioral addictions have early-onset rates (in late adolescence). In addition, they are done willingly and can be controlled initially, but in later stages they might become compulsive (3, 4). Third, these behaviors have the capacity to alter one's different moods to a euphoric status by performing the behavior; however, they need to increase the frequency and duration of the behavior over time to achieve this euphoric feeling, which is similar to the definition of tolerance (3). Finally, neurobiological changes, such as reduced D2-like receptor in brain regions involved with reward circuit, were found in compulsive internet users (5). Moreover, neuropathological alterations have been documented with internet gaming disorder and pornography addiction (6, 7).

Internet addiction is characterized as a strong irresistible desire to constantly use the internet. Also, it exhibits intolerance, which leads to increase in the duration of usage overtime to the degree that it damages the user psychologically and socially and that it shows somatic symptoms (4). It also has been associated with attention-deficit/hyperactivity disorder and suicidal thoughts (8–10). Despite these findings in the literature, the (DSM-V) (2013) and the International Classification of Diseases The 11th Revision (ICD-11) (2019) have not categorized internet addiction as a disorder yet (11).

One of the common behavioral addictions is video gaming addiction, which has been proposed in (DSM-V) (2013) as a potential disorder and was classified in the (ICD-11) (2019) as a behavioral disorder. Video-gaming addiction is a behavioral addiction characterized by loss of control and functional impairment. Furthermore, it has been associated with low well-being, negative consequences, and other internet addictions (6, 7, 12).

Another discussed behavioral addiction is Compulsive sexual behavior disorder (CSBD), which is a controversial and sensitive subject. According to (ICD-11) (2019), CSBD is characterized by a persistent pattern of failure to control intense, repetitive sexual impulses, or urges resulting in repetitive sexual behaviors. Internet pornography addiction, also known as problematic pornography use is a subtype of CSBD that is associated with some clinical manifestations including erectile dysfunction, psychosexual dissatisfaction, and many co-morbidities such as anxiety, mood disorders, and sexual dysfunction (13). Moreover, neurological data suggest strong evidence for considering problematic pornography use as a behavioral addiction (14). Besides, pornography addiction has been found to be the second most common behavioral addiction among males in Ontario, Canada (15).

Finally, stress and anxiety have been linked to various forms of addiction, including behavioral addictions (2, 16, 17). Researches have revealed that medical students are susceptible to stress and anxiety. In fact, it has been found that more than half of the medical students experience stress (17). A local study done at the University of King Abdulaziz showed that 34% of female medical students have anxiety related to their major at school (18).

The prevalence of behavioral addictions is not well-established in Saudi Arabia. In addition, the association between stress and anxiety with behavioral addictions has not been fully explored. The aim of this study is to measure the prevalence of internet, online video-gaming, and pornography addictions among medical students in Western region. Also, we intend to determine the relationship between these behavioral addictions with stress and anxiety.

## Methods

### Ethical Approval

This study was approved by King Abdullah International Medical Research Center (KAIMRC) institutional review board. Study number: SP20/063/J.

### Design and Setting

This study is an observational cross-sectional online questionnaire-based study. The study targeted medical students in their 3rd, 4th, and 5th academic years from five different medical colleges in the Western region of the kingdom of Saudi Arabia. The selection of participants was through the quota sampling technique. The questionnaires' links were sent to each college students' (WhatsApp) group. To ensure correct representation from each college, different links were sent to each individual college. However, the composition of the questionnaire provided was identical across all links. Following the distribution of the questionnaire through links provided in WhatsApp groups, participants had the option to voluntarily participate or decline. Moreover, participants could view the content of the link before clicking and participating as a brief introduction of the aims and objectives of the study is displayed alongside the link provided in the WhatsApp groups. After clicking on the link, participants can review the aims and objects of the study again and the authors involved. Following that, they will need to confirm the participation in the study and choose either “agree” to continue with the study or “disagree” and in either case, the participants' identity will remain anonymous. The data was collected during the period of December 2019 to February 2020. Also, each college received a link to ensure an accurate representation of all medical students among the five universities. Desirable sample size was calculated to be 328 through Sample Size Calculator by Raosoft, Inc. A confidence interval of 95% was implanted for the calculation, with a margin of error of 5%, population proportion of 50%, and estimated total population of 2,200.

### Questionnaires

Google's Forms was used to form the questionnaire and collect data. The questionnaire was composed of seven sections. The first section included a brief introduction about the study and showed the informed consent form with option to proceed or leave the questionnaire. The second section assessed the demographics of the participants (age, gender, academic year, income, marital status), and sections three to seven included the used scales. A full copy of the questionnaire can be accessed in the Supplementary Material. Four independent data collectors sent the forms to the participants, and they sent the responses to the authors.

### Behavioral Assessment Scales

The questionnaire included five validated scales: Young's Internet Addiction Test—Short Version (IAT-SV) (19), Internet Gaming Disorder Scale 9—Short Form (IGDS9-SF) (20), the Problematic Pornography Consumption Scale (PPCS) (21), Perceived Stress Scale (PSS) (22), and Generalized Anxiety Disorder 7-item scale (GAD-7) (23). Each one of those scales is reported as a continuous score, which was converted to categorical outcomes. No changes were made on scales scoring criteria and categories cut-off points, which are provided by each scale's corresponding author.

Three scales (IAT-SV, IGDS9-SF, PPCS) were used to assess specific areas of behavioral addictions. The other two scales (PSS, GAD-7) were utilized to assess participants' levels of stress and anxiety.

### Data Analysis

Data entry was conducted by the researchers on an Excel file. Afterwards, data were transferred to Statistical Package for the Social Sciences (SPSS) software version 20.0 for analysis. Mean and standard deviation were used to describe variables that are normally distributed, while median and interquartile range were used to describe variables that are skewed. Shapiro-Wilk test was used to assess the normality. To estimate the association between behavioral addictions and stress and anxiety, the chi-square test was used (fisher's exact test was used when the event rate was 5 or less). Furthermore, the scales' scores were analyzed using Spearman's Correlation Test to assess the correlation between behavioral addictions with stress and anxiety. Eta squared for variables, small: college year and marital status, medium: gender, income GAD, PPC, PSS, and IGD, and finally large: IAT. The regression model has been done to highlight the significant outcomes (Supplementary Material). Statistical significance was considered at a *P* < 0.05.

## Results

The total number of participants was 225, which is 68.5% out of the 328 calculated sample size. The median age of participants was 21.00. The number of male participants was 136 (60.4%) and the number of female participants was 89 (39.6%). The participants were divided as follows: 77 (43.2%) in the 3rd year, 108 (48%) in the 4th year, and 40 (17.8%) in the 5th year (Table 1).

**Table 1:** Demographics and prevalence of outcomes.

	***n***	**%**
**Gender**		
Male	136	60.4
Female	89	39.6
**College year**		
3rd Year	77	34.2
4th Year	108	48.0
5th Year	40	17.8
**Marital status**		
Married	5	2.2
Non-married	220	97.8
**Household income**		
<5,000 SR	27	12.0
5,000–10,000 SR	35	15.6
10,000–15,000 SR	35	15.6
More than 15,000 SR	128	56.9
**Internet addiction test result**		
Normal	71	31.6
Problematic	51	22.7
Pathological	103	45.8
**Generalized anxiety disorder result**		
Normal	152	67.6
Moderate anxiety	50	22.2
Severe anxiety	23	10.2
**Problematic pornography consumption result**		
Normal	199	88.4
Possible	26	11.6
**Perceived stress scale result**		
Low stress	35	15.6
Moderate stress	146	64.9
High stress	44	19.6
**Internet gaming disorder result**		
None disordered	220	97.8
Disordered	5	2.2
Total	225	100.0

The prevalence of stress was measured by (PSS): low stress in 35 (15.6%), moderate stress in 146 (46.9%), and high stress in 44 (19.6%). Moving on, anxiety was found by (GAD-7) to be normal in 152 (67.6%), moderate anxiety in 50 (22.2%), and severe anxiety in 23 (10.2%). Furthermore, the IAT-SV scale showed normal internet usage in 71 (31.6%), problematic in 51 (22.7%), and pathologic in 103 (45.8%). The IGDS9-SF scale was used to measure internet gaming disorder, and the following values were observed according to the scales: 220 (97.8%) were non-disordered, and 5 (2.2%) were found to be disordered. The PPCS scale was used with a cutoff point of 76. The scale had shown that 199 (88.4%) were normal, and 26 (11.6%) had possible problematic use (Table 1).

Only IAT-SV scores were normally distributed, the rest of the scales' scores were skewed. Thus, IAT-SV scores mean was 34.91. The medians were as follow; PSS: 20.00, GAD-7: 7.00, PPCS: 30.00, and IGDS9-SF: 12.00 (Table 2).

**Table 2:** Scores of different scales.

**Normally distributed results**				
			**95% confidence interval**	
	**Mean**	**s.d**	**Lower bound**	**Upper bound**
**Internet addiction test**				
	34.91	8.04	33.86	35.97
**Skewed results**				
	**Median**	**Minimum**	**Maximum**	**Interquartile range**
**Age**				
	21.00	18.00	30.00	2.00
**Perceived stress scale**				
	20.00	4.00	37.00	8.00
**Generalized anxiety disorder**				
	7.00	0.00	21.00	9.00
**Problematic pornography consumption**				
	30.00	18.00	106.00	37.00
**Internet gaming disorder**				
	12.00	9.00	45.00	13.00

*Shapiro-Wilk test was used to assess the normality*.

As illustrated in Table 3, there was no significant association between stress and gender (*P*-value 0.713), college year (*P*-value = 0.71), and internet gaming disorder (*P*-value = 0.41). On the contrary, statistical analysis showed a highly significant association between stress and problematic pornography consumption (*P*-value = 0.01), and internet addiction (*P* < 0.001). Moreover, the association of anxiety and the study variables are listed in Table 4. Statistical analysis also revealed a significant association between anxiety and the gender of participants (*P*-value = 0.02). Additionally, there was a highly significant association between anxiety and problematic pornography use (*P* < 0.001). Moreover, there was a significant association between anxiety and internet gaming disorder (*P*-value = 0.01) and Internet disorder (*P*-value = 0.01). However, there was no significant association between college year and anxiety (*P*-value = 0.37).

**Table 3:** Association between different behavioral addictions and stress.

		**Low stress**		**Moderate stress**		**High stress**		**Total**		***P*** **-value**
		***n***	**%**	***n***	**%**	***n***	**%**	***n***	**%**	
**Gender**										0.36
	**Male**									
		20	57.1	93	63.7	23	52.3	136	60.4	
	**Female**									
		15	42.9	53	36.3	21	47.7	89	39.6	
	**Total**									
		35	100.0	146	100.0	44	100.0	225	100.0	
**College year**										0.71
	**3rd year**									
		11	14.3	50	64.9	16	20.8	77	100.0	
	**4th year**									
		20	18.5	67	62.0	21	19.4	108	100.0	
	**5th year**									
		4	10.0	29	72.5	7	17.5	40	100.0	
	**Total**									
		35	15.6	146	64.9	44	19.6	225	100.0	
**Generalized anxiety disorder**										<0.001
	**Normal**									
		35	100.0	107	73.3	10	22.7	152	67.6	
	**Moderate anxiety**									
		0	0.0	31	21.2	19	43.2	50	22.2	
	**Severe anxiety**									
		0	0.0	8	5.5	15	34.1	23	10.2	
	**Total**									
		35	100.0	146	100.0	44	100.0	225	100.0	
**Problematic pornography consumption**										0.02
	**Normal**									
		34	97.1	131	89.7	34	77.3	199	88.4	
	**Possible**									
		1	2.9	15	10.3	10	22.7	26	11.6	
	**Total**									
		35	100.0	146	100.0	44	100.0	225	100.0	
**Internet gaming disorder**										0.41^*^
	**Non-disordered**									
		35	100.0	143	97.9	42	95.5	220	97.8	
	**Disordered**									
		0	0.0	3	2.1	2	4.5	5	2.2	
	**Total**									
		35	100.0	146	100.0	44	100.0	225	100.0	
**Internet addiction test**										<0.001
	**Normal**									
		21	60.0	43	29.5	7	15.9	71	31.6	
	**Problematic**									
		11	31.4	36	24.7	4	9.1	51	22.7	
	**Pathological**									
		3	8.6	67	45.9	33	75.0	103	45.8	
	**Total**									
		35	100.0	146	100.0	44	100.0	225	100.0	

*Pearson Chi-Square was used to calculate p-value*.

* *For internet gaming disorder, Fisher's exact test was used to calculate p-value*.

**Table 4:** Association between different behavioral addictions and anxiety.

		**Normal anxiety**		**Moderate anxiety**		**Severe anxiety**		**Total**		***P*** **-value**
		***n***	**%**	***n***	**%**	***n***	**%**	***n***	**%**	
**Gender**										0.02
	**Male**									
		93	61.2	35	70.0	8	34.8	136	60.4	
	**Female**									
		59	38.8	15	30.0	15	65.2	89	39.6	
	**Total**									
		152	100.0	50	100.0	23	100.0	225	100.0	
**College year**										0.37
	**3rd year**									
		47	30.9	22	44.0	8	34.8	77	34.2	
	**4th year**									
		78	51.3	21	42.0	9	39.1	108	48.0	
	**5th year**									
		27	17.8	7	14.0	6	26.1	40	17.8	
	**Total**									
		152	100.0	50	100.0	23	100.0	225	100.0	
**Perceived stress scale**										<0.001
	**Low stress**									
		35	23.0	0	0.0	0	0.0	35	15.6	
	**Moderate stress**									
		107	70.4	31	62.0	8	34.8	146	64.9	
	**High stress**									
		10	6.6	19	38.0	15	65.2	44	19.6	
	**Total**									
		152	100.0	50	100.0	23	100.0	225	100.0	
**Problematic pornography consumption**										<0.001
	**Normal**									
		146	96.1	34	68.0	19	82.6	199	88.4	
	**Possible**									
		6	3.9	16	32.0	4	17.4	26	11.6	
	**Total**									
		152	100.0	50	100.0	23	100.0	225	100.0	
**Internet gaming disorder**										0.01^*^
	**Non-disordered**									
		151	99.3	49	98.0	20	87.0	220	97.8	
	**Disordered**									
		1	0.7	1	2.0	3	13.0	5	2.2	
	**Total**									
		152	100.0	50	100.0	23	100.0	225	100.0	
**Internet addiction test**										0.01
	**Normal**									
		55	36.2	11	22.0	5	21.7	71	31.6	
	**Problematic**									
		40	26.3	8	16.0	3	13.0	51	22.7	
	**Pathological**									
		57	37.5	31	62.0	15	65.2	103	45.8	
	**Total**									
		152	100.0	50	100.0	23	100.0	225	100.0	

*Pearson Chi-Square was used to calculate p-value*.

* *For internet gaming disorder, Fisher's exact test was used to calculate p-value*.

**Figure 1:**
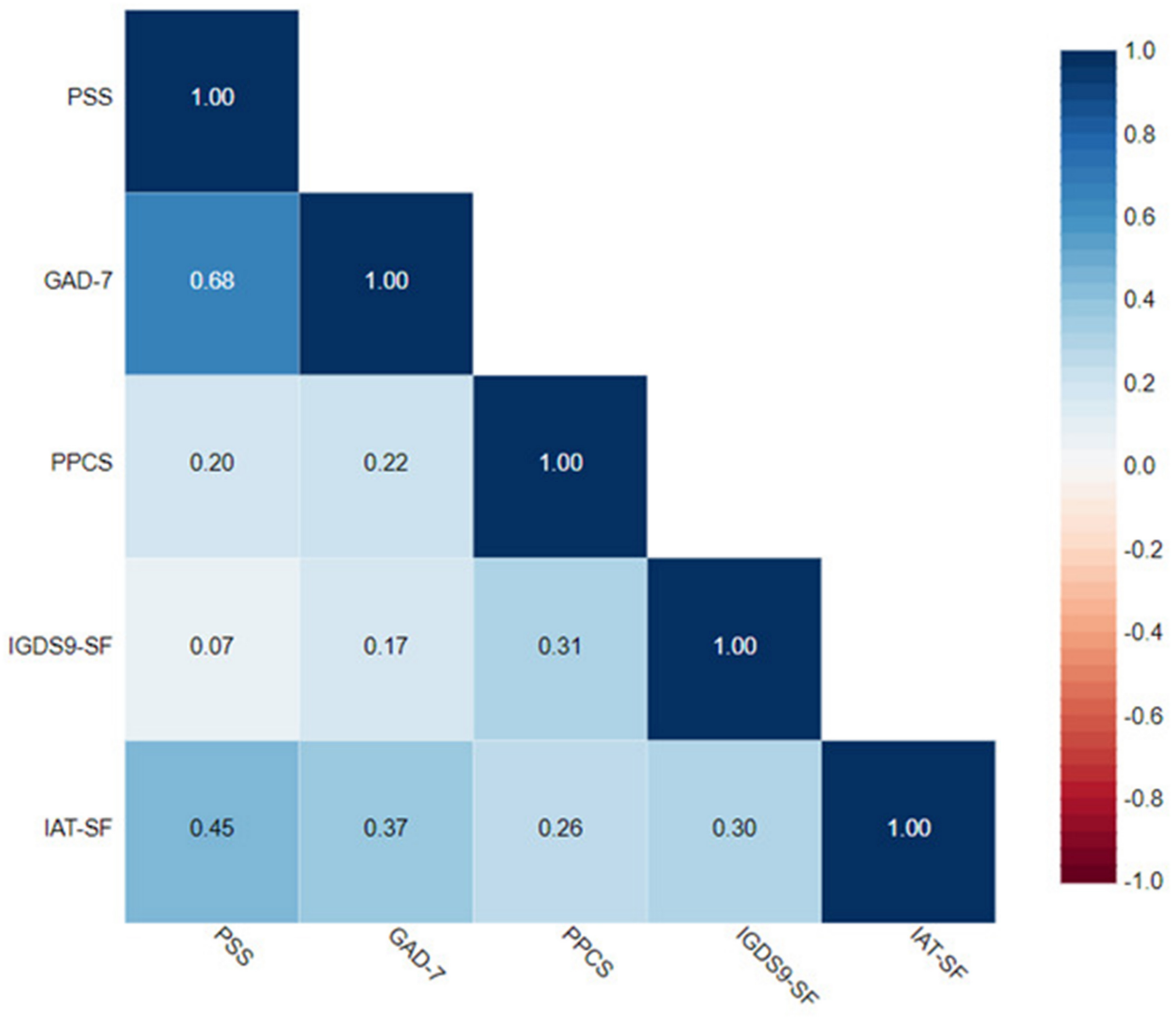
Correlation matrix.

Statistical analysis exhibited a moderate correlation between GAD-7 and PSS with *r* = 0.68. Moreover, there was also a moderate correlation between GAD-7 and IAT-SV with *r* = 0.38. PSS was found to have a moderate correlation with IGDS9-SF with *r* = 0.45. In addition, PPCS was found to have a moderate correlation with IAT-SV with *r* = 0.31. Furthermore, there was a moderate correlation between IGDS9-SF and IAT-SV with *r* = 0.30 (Figure 1).

## Discussion

This study aims to measure the prevalence of certain behavioral addictions including internet addiction, video gaming addiction, and problematic pornography use. Also, this study investigated the associations between these behavioral addictions with stress and anxiety. In addition, these types of behavioral addiction have a cultural sensitivity aspect. Therefore, in order to receive a rapid and non-biased response, non-invasive tools such as anonymous questionnaires are more suitable and provide higher reliability outcomes. The outcomes of this study might be helpful for the policymakers to assess the problems of the society timely and make necessary recommendations. The prevalence of internet addiction in medical students at different colleges in Western region was high (45.8%), with (22.7%) of the students showing problematic internet behavior. In comparison with a meta-analysis, the pooled prevalence was found to be (30.1%) in medical students (24). Nonetheless, both studies showed a high prevalence of internet addiction in medical students. Moreover, behavioral addiction is different than substance abuse in the availability of guidance, treatment, or rehabilitation center. Thus, this meta-analysis revealed some of the recommendations to attenuate the addictive use of the internet: (a) Internet use in opposite time practice (discover participants patterns of Internet use and propose a new schedule to disrupt the addiction patterns), (b) Suggest including external activities to stop the addiction patterns (real events or activities encouraging the patient to log off), (c) Involvement of previously determined goals regarding the amount of time, (d) Identify the applications, which the participant is unable to control and manage the time spent on the application, and (e) Reminder cards technique with the costs of internet addiction and the benefits of breaking addiction habit (9). Furthermore, the prevalence of internet gaming disorder was found to be low (2.2%). A systematic review done on 28 studies from across the globe exhibited a slightly higher pooled value of (4.6%) (25). This difference might be because of the different age groups of the participants between the two samples. For example, our sample solely had adults compared with the systematic review which focused on adolescents aged 10–19 years (25). In addition, problematic pornography use had a prevalence of (11.6%) in our study which is considered similar to other studies that found it to be (10%) and (12.2%) (26, 27).

Our study revealed that there was a significant association between stress and internet addiction among medical students. Comparable results were found by another study conducted on Lebanese college students where a strong correlation was found between internet addiction and anxiety, stress, and depression (28). Furthermore, our study demonstrated an association between stress and problematic pornography use. To the best of our knowledge, there is a lack of research focused on this association. On the contrary, statistical analysis found no association between stress and internet gaming disorder. In contrast to our results, a recent study has found that there was an association between stress and internet gaming disorder (29). The reason behind these differences might be attributed to a different collection method in which an interview method was used in the latter study. Also, our sample population was medical students in contrast to young adults in the mentioned study could be also due to the low prevalence of internet gaming disorder among our sample. Statistical analysis exhibited a significant association between anxiety and internet addiction, although we had small sample size, which was in similar outcomes to what was reported by Younes et al. (28). Additionally, our results suggested an association between anxiety and internet gaming disorder. The results in our study were in line with a study reported by Fazeli et al. (30). Previous studies have investigated the impact of the internet addiction on affective dysregulation. A study found that participants with higher score on internet addiction were more likely to report greater difficulties related to affective regulation (31). Importantly, a study suggests that the prefrontal-limbic circuit may play a major role in affective dysregulation, as well as the insula, the dorsal anterior cingulate cortex, and prefrontal areas involved in the cognitive regulation of emotions and enhancing the coupling of limbic and prefrontal areas. When activated, dopamine release is increased, along with opiates and other neurochemicals. With chronic effect, the associated receptors may be affected, producing tolerance or the need for increased stimulation of the reward center to produce a “high” and the subsequent characteristic behavior pattern needed to avoid withdrawal symptoms. (32). A previous study was conducted on 1,086 engineering students from India, has used the socio-educational and internet use behavior data sheet to investigate the demographic information and internet use patterns (33). This latter study has revealed that 27.1% of the student's met criterion for mild internet addiction. In addition, ~10 and 0.5% of the students met the criterion for moderate and severe internet addiction, respectively.

## Limitations and Future Directions

The study outcomes may assist in developing a method for the intervention of behavioral addiction and raising population awareness. Moreover, the availability of consultation services rather than rehabilitation centers for the population will contribute to alleviating the potential impact of this addiction. This study had certain limitations starting with the COVID-19 pandemic, which restricted and changed our data collection method. The study reporting was intended to be through a paper-based questionnaire. However, an internet-based survey was used as an alternative way to collect the responses due to this unprecedented situation. Consequently, our sample size did not reach the calculated desired number due to the virtual transformation of the educational process, and therefore, it was more difficult to approach the participants. Importantly, the sensitivity of behavioral addictions in most cultures (including our culture) might be a contributor to the low response rate in the study. Thus, the results of this study might lead to an underestimation of the actual prevalence of these addictions. Further studies are guaranteed to recruit more participants, investigate the issues in the general population and with different age groups. For human models, many confounding factors can contribute to the effect, especially regarding the aspects of behavioral addiction. These factors include the socioeconomic factors and the family-bonding effects (strong or weak family bonds). Moreover, peer pressure can be another confounding factor to be included. Further investigation is required to reveal the effect of those confounding factors as they might significantly impact behavioral addiction.

## Conclusion

With the continuous growth of internet and easy accessibility to videogames and pornography, addiction to them will be a major concern. Our results showed a high prevalence of behavioral addiction especially internet addiction and problematic pornography use, and indicated the possible association with stress and anxiety. To sum up, we expect the problem only to increase over times. Therefore, more studies are needed to confirm the prevalence and to examine the causation/association relationship with stress and anxiety. Moreover, further studies are guaranteed to recruit more participants, investigate the issues in the general population and with different age groups. Finally, the effect of these behavioral addictions on quality of life and function needs to be assessed to guide appropriate interventions.

## Data Availability Statement

The raw data supporting the conclusions of this article will be made available by the authors, without undue reservation.

## Ethics Statement

The studies involving human participants were reviewed and approved by King Abdullah International Medical Research Center (KAIMRC) Institutional Review Board. The patients/participants provided their written informed consent to participate in this study.

## Author Contributions

All authors listed have made substantial, direct, and intellectual contribution to the work and approved it for publication.

## Conflict of Interest

The authors declare that the research was conducted in the absence of any commercial or financial relationships that could be construed as a potential conflict of interest.

## Publisher's Note

All claims expressed in this article are solely those of the authors and do not necessarily represent those of their affiliated organizations, or those of the publisher, the editors and the reviewers. Any product that may be evaluated in this article, or claim that may be made by its manufacturer, is not guaranteed or endorsed by the publisher.
