# Supplementary material for: Prevalence of Behavioral Addictions and Their Relationship With Stress and Anxiety Among Medical Students in Saudi Arabia: A Cross-Sectional Study
Source: Front Psychiatry. 2021 Aug 17;12:727798. doi: 10.3389/fpsyt.2021.727798 (PMC8416092; doi:10.3389/fpsyt.2021.727798)
Supplement: Supplementary file 1 [file Data_Sheet_1.pdf]

A Questionnaire-Based Survey to Asses The Prevalence of Behavioral Addictions among Medical Students in Jeddah

*Required

You are requested to participate in a research that will be supervised by (Dr. Al-Qassim Hakami) (King Saud Bin Abdulaziz University For Health Sciences, Jeddah). The aim of the study is to measure the prevalence of behavioral addiction among medical students and its asscoiation with stress and anxiety. The information collected from this survey will be handle with utmost confidentiality and will only be used for the research purposes.

Research Supervisor's Email: Hakamia@ksau-hs.edu.sa

Demographics

1. Age *

____

2. Gender *

Mark only one oval.

- ( ) Male
- ( ) Female

3. College Year *

Mark only one oval.

- ( ) 3rd
- ( ) 4th
- ( ) 5th

4. Marital Satus *

Mark only one oval.

- Married
- Not Married

5. Household Income *

Mark only one oval.

- Less than 5,000 SR
- 5,000-10,000 SR
- 10,000-15,000 SR
- More than 15,000 SR

Young’s Internet Addiction Test

Consider only non-academic or recreational internet use.

6. 1. How often do you find that you stay on-line longer than you intended? *

Tick all that apply.

	Never	Rarely	Sometimes	Often	Very Often
الصف 1	☐	☐	☐	☐	☐

7. 2. How often do you neglect household chores to spend more time on-line? *

Mark only one oval.

- Never
- Rarely
- Sometimes
- Often
- Very Often

8. 3. How often do your grades or school work suffer because of the amount of time you spend on-line? *

Mark only one oval.

- Never
- Rarely
- Sometimes
- Often
- Very Often

9. 4. How often do you become defensive or secretive when anyone asks you what you do on-line? *

Mark only one oval.

- Never
- Rarely
- Sometimes
- Often
- Very Often

10. 5. How often do you snap, yell, or act annoyed if someone bothers you while you are on-line *

Mark only one oval.

- Never
- Rarely
- Sometimes
- Often
- Very Often

11. 6. How often do you lose sleep due to being online late at night? *

Mark only one oval.

- Never
- Rarely
- Sometimes
- Often
- Very Often

12. 7. How often do you feel preoccupied with the Internet when off-line, or fantasize about being on-line? *

Mark only one oval.

- Never
- Rarely
- Sometimes
- Often
- Very Often

13. 8. How often do you find yourself saying “just a few more minutes” when on-line? *

Mark only one oval.

- Never
- Rarely
- Sometimes
- Often
- Very Often

14. 9. How often do you try to cut down the amount of time you spend on-line and fail? *

Mark only one oval.

- Never
- Rarely
- Sometimes
- Often
- Very Often

15. 10. How often do you try to hide how long you've been on-line? *

Mark only one oval.

- Never
- Rarely
- Sometimes
- Often
- Very Often

16. 11. How often do you choose to spend more time on-line over going out with others? *

Mark only one oval.

- Never
- Rarely
- Sometimes
- Often
- Very Often

17. 12. How often do you feel depressed, moody, or nervous when you are off-line, which goes away once you are back on-line? *

Mark only one oval.

- Never
- Rarely
- Sometimes
- Often
- Very Often

Generalized Anxiety Disorder 7-item (GAD-7) scale

Over the last 2 weeks, how often have you been bothered by the following problems?

18. 1. Feeling nervous, anxious, or on edge *

Mark only one oval.

- Not at all sure
- Several days
- Over half the days
- Nearly every day

19. 2. Not being able to stop or control worrying *

Mark only one oval.

- Not at all sure
- Several days
- Over half the days
- Nearly every day

20. 3. Worrying too much about different things *

Mark only one oval.

- Not at all sure
- Several days
- Over half the days
- Nearly every day

21. 4. Trouble relaxing *

Mark only one oval.

- Not at all sure
- Several days
- Over half the days
- Nearly every day

22. 5. Being so restless that it's hard to sit still *

Mark only one oval.

- Not at all sure
- Several days
- Over half the days
- Nearly every day

23. 6. Becoming easily annoyed or irritable *

Mark only one oval.

- Not at all sure
- Several days
- Over half the days
- Nearly every day

24. 7. Feeling afraid as if something awful might happen *

Mark only one oval.

- Not at all sure
- Several days
- Over half the days
- Nearly every day

Internet Gaming Disorder Scale 9 – Short Form (IGDS9-SF)

These questions will ask you about your gaming activity during the past year (i.e., last 12 months). By gaming activity we understand any gaming-related activity that has been played either from a computer/laptop or from a gaming console or any other kind of device (e.g., mobile phone, tablet, etc.) both online and/or offline.

25. 1. Do you feel preoccupied with your gaming behavior? (Some examples: Do you think about previous gaming activity or anticipate the next gaming session? Do you think gaming has become the dominant activity in your daily life?) *

Mark only one oval.

- Never
- Rarely
- Sometimes
- Often
- Very Often

26. 2. Do you feel more irritability, anxiety or even sadness when you try to either reduce or stop your gaming activity? *

Mark only one oval.

- Never
- Rarely
- Sometimes
- Often
- Very Often

27. 3. Do you feel the need to spend increasing amount of time engaged gaming in order to achieve satisfaction or pleasure? *

Mark only one oval.

- Never
- Rarely
- Sometimes
- Often
- Very Often

28. 4. Do you systematically fail when trying to control or cease your gaming activity? *

Mark only one oval.

- Never
- Rarely
- Sometimes
- Often
- Very Often

29. 5. Have you lost interests in previous hobbies and other entertainment activities as a result of your engagement with the game? *

Mark only one oval.

- Never
- Rarely
- Sometimes
- Often
- Very Often

30. 6. Have you continued your gaming activity despite knowing it was causing problems between you and other people? *

Mark only one oval.

- Never
- Rarely
- Sometimes
- Often
- Very Often

31. 7. Have you deceived any of your family members, therapists or others because the amount of your gaming activity? *

Mark only one oval.

- Never
- Rarely
- Sometimes
- Often
- Very Often

32. 8. Do you play in order to temporarily escape or relieve a negative mood (e.g., helplessness, guilt, anxiety)? *

Mark only one oval.

- Never
- Rarely
- Sometimes
- Often
- Very Often

33. 9. Have you jeopardized or lost an important relationship, job or an educational or career opportunity because of your gaming activity? *

Mark only one oval.

- Never
- Rarely
- Sometimes
- Often
- Very Often

Perceived Stress Scale

The questions in this scale ask you about your feelings and thoughts during THE LAST MONTH. In each case, please indicate your response by placing an "X" over the circle representing HOW OFTEN you felt or thought a certain way.

34. 1. In the last month, how often have you been upset because of something that happened unexpectedly? *

Mark only one oval.

- Never
- Almost Never
- Sometimes
- Fairly Often
- Very Often

35. 2. In the last month, how often have you felt that you were unable to control the important things in your life? *

Mark only one oval.

- Never
- Almost Never
- Sometimes
- Fairly Often
- Very Often

36. 3. In the last month, how often have you felt nervous and “stressed”? *

Mark only one oval.

- Never
- Almost Never
- Sometimes
- Fairly Often
- Very Often

37. 4. In the last month, how often have you felt confident about your ability to handle your personal problems? *

Mark only one oval.

- Never
- Almost Never
- Sometimes
- Fairly Often
- Very Often

38. 5. In the last month, how often have you felt that things were going your way? *

Mark only one oval.

- Never
- Almost Never
- Sometimes
- Fairly Often
- Very Often

39. 6. In the last month, how often have you found that you could not cope with all the things that you had to do? *

Mark only one oval.

- Never
- Almost Never
- Sometimes
- Fairly Often
- Very Often

40. 7. In the last month, how often have you been able to control irritations in your life? *

Mark only one oval.

- Never
- Almost Never
- Sometimes
- Fairly Often
- Very Often

41. 8. In the last month, how often have you felt that you were on top of things? *

Mark only one oval.

- Never
- Almost Never
- Sometimes
- Fairly Often
- Very Often

42. 9. In the last month, how often have you been angered because of things that were outside your control? *

Mark only one oval.

- Never
- Almost Never
- Sometimes
- Fairly Often
- Very Often

43. 10. In the last month, how often have you felt difficulties were piling up so high that you could not overcome them? *

Mark only one oval.

- Never
- Almost Never
- Sometimes
- Fairly Often
- Very Often

The Problematic Pornography Consumption Scale (PPCS)

Please think back to the past six months and indicate on the following 7-point scale how often or to what extent the statements apply to you. There is no right or wrong answer. Please indicate the answer that most applies to you.

44. 1. I felt that porn is an important part of my life *

Mark only one oval.

- Never
- Rarely
- Occasionally
- Sometimes
- Often
- Very Often
- All the time

45. 2. I used porn to restore the tranquility of my feelings *

Mark only one oval.

- Never
- Rarely
- Occasionally
- Sometimes
- Often
- Very Often
- All the time

46. 3. I felt porn caused problems in my sexual life *

Mark only one oval.

- Never
- Rarely
- Occasionally
- Sometimes
- Often
- Very Often
- All the time

47. 4. I felt that I had to watch more and more porn for satisfaction *

Mark only one oval.

- Never
- Rarely
- Occasionally
- Sometimes
- Often
- Very Often
- All the time

48. 5. I unsuccessfully tried to reduce the amount of porn I watch *

Mark only one oval.

- Never
- Rarely
- Occasionally
- Sometimes
- Often
- Very Often
- All the time

49. 6. I became stressed when something prevented me from watching porn *

Mark only one oval.

- Never
- Rarely
- Occasionally
- Sometimes
- Often
- Very Often
- All the time

50. 7. I thought about how good it would be to watch porn *

Mark only one oval.

- Never
- Rarely
- Occasionally
- Sometimes
- Often
- Very Often
- All the time

51. 8. Watching porn got rid of my negative feelings *

Mark only one oval.

- Never
- Rarely
- Occasionally
- Sometimes
- Often
- Very Often
- All the time

52. 9. Watching porn prevented me from bringing out the best in me *

Mark only one oval.

- Never
- Rarely
- Occasionally
- Sometimes
- Often
- Very Often
- All the time

53. 10. I felt that I needed more and more porn in order to satisfy my needs *

Mark only one oval.

- Never
- Rarely
- Occasionally
- Sometimes
- Often
- Very Often
- All the time

54. 11. When I vowed not to watch porn anymore, I could only do it for a short period of time *

Mark only one oval.

- Never
- Rarely
- Occasionally
- Sometimes
- Often
- Very Often
- All the time

55. 12. I became agitated when I was unable to watch porn *

Mark only one oval.

- Never
- Rarely
- Occasionally
- Sometimes
- Often
- Very Often
- All the time

56. 13. I continually planned when to watch porn *

Mark only one oval.

- Never
- Rarely
- Occasionally
- Sometimes
- Often
- Very Often
- All the time

57. 14. I released my tension by watching porn *

Mark only one oval.

- Never
- Rarely
- Occasionally
- Sometimes
- Often
- Very Often
- All the time

58. 15. I neglected other leisure activities as a result of watching porn *

Mark only one oval.

- Never
- Rarely
- Occasionally
- Sometimes
- Often
- Very Often
- All the time

59. 16. I gradually watched more “extreme” porn, because the porn I watched before was less satisfying *

Mark only one oval.

- Never
- Rarely
- Occasionally
- Sometimes
- Often
- Very Often
- All the time
