# Supplementary material for: Prevalence of Behavioral Addictions and Their Relationship With Stress and Anxiety Among Medical Students in Saudi Arabia: A Cross-Sectional Study
Source: Front Psychiatry. 2021 Aug 17;12:727798. doi: 10.3389/fpsyt.2021.727798 (PMC8416092; doi:10.3389/fpsyt.2021.727798)
Supplement: Supplementary file 2 [file Table_1.docx]

**Supplementary table 1: Logistic regression of Stress with different factors**

|  | **Adjusted OR** | **95% CI** | | **p** |
| --- | --- | --- | --- | --- |
| KSAU-HS | 1 |  |  | 0.590 |
| KAU | 4.69 | 0.72-30.62 | | 0.106 |
| Jeddah University | 4.13 | 0.53-32.05 | | 0.175 |
| Ibn Sina University | 0.00 | 0.00 | | 0.997 |
| Batarjee University | 2.70 | 0.26-28.12 | | 0.407 |
| Age | 1.45 | 0.96-2.19 | | 0.081 |
| Male | 1.21 | 0.38-3.82 | | 0.745 |
| Female | 1 |  | |  |
| 3rd Year | 1 |  |  | 0.476 |
| 4th Year | 1.73 | 0.31-9.75 | | 0.534 |
| 5th Year | 0.80 | 0.16-3.95 | | 0.780 |
| Married | 7.83 | 0.38-162.82 | | 0.184 |
| Non-Married | 1 |  | |  |
| < 5000 SR | 1 |  |  | 0.776 |
| 5000-10000 SR | 0.73 | 0.18-3.01 | | 0.662 |
| 10001-15000 SR | 0.50 | 0.09-2.83 | | 0.430 |
| > 15000 SR | 0.63 | 0.20-2.01 | | 0.435 |
| Normal | 1 |  |  | 0.005 |
| Problematic | 0.10 | 0.02-0.40 | | 0.001 |
| Pathological | 0.15 | 0.03-0.64 | | 0.010 |
| Normal | 1.38 | 0.11-16.84 | | 0.799 |
| Possible | 1 |  | |  |
| None Disordered | 31119669 | 0.00 | | 0.999 |
| Disordered | 1 |  | |  |
| Normal | 1 |  |  | >0.999 |
| Moderate Anxiety | 0.00 | 0.00 | | 0.998 |
| Severe Anxiety | 1162800 | 0.00 | | 0.999 |
