# Supplementary material for: Prevalence of Behavioral Addictions and Their Relationship With Stress and Anxiety Among Medical Students in Saudi Arabia: A Cross-Sectional Study
Source: Front Psychiatry. 2021 Aug 17;12:727798. doi: 10.3389/fpsyt.2021.727798 (PMC8416092; doi:10.3389/fpsyt.2021.727798)
Supplement: Supplementary file 3 [file Table_2.docx]

**Supplementary table 2: Logistic regression of Stress with different factors**

|  | **Adjusted OR** | **95% CI** | | **p** |
| --- | --- | --- | --- | --- |
| KSAU-HS | 1 |  |  | 0.358 |
| KAU | 3.52 | 0.36-34.28 | | 0.278 |
| Jeddah University | 1.06 | 0.08-13.50 | | 0.963 |
| Ibn Sina University | 616315082 | 0.00 | | 0.997 |
| Batarjee University | 1.95 | 0.19-20.42 | | 0.578 |
| Age | 0.76 | 0.56-1.03 | | 0.081 |
| Male | 0.86 | 0.33-2.20 | | 0.750 |
| Female | 1 |  | |  |
| 3rd Year | 1 |  |  | 0.763 |
| 4th Year | 0.96 | 0.25-3.72 | | 0.957 |
| 5th Year | 0.70 | 0.21-2.30 | | 0.556 |
| Married | 0.70 | 0.03-16.65 | | 0.825 |
| Unmarried | 1 |  | |  |
| <5000 SR | 1 |  |  | 0.026 |
| 5000-10000 SR | 0.97 | 0.26-3.65 | | 0.966 |
| 10001-15000 SR | 5.93 | 1.78-19.76 | | 0.004 |
| >15000 SR | 1.29 | 0.37-4.43 | | 0.687 |
| Normal | 1 |  |  | 0.838 |
| Problematic | 0.87 | 0.33-2.31 | | 0.782 |
| Pathological | 0.72 | 0.24-2.14 | | 0.556 |
| Normal (PPC) | 8.40 | 2.03-34.75 | | 0.003 |
| Low stress | 1 |  |  | <0.001 |
| Moderate stress | 0.00 | 0.00 | | 0.996 |
| High stress | 0.08 | 0.03-0.23 | | <0.001 |
| None disordered (IGD) | 1.21 | 0.05-30.11 | | 0.907 |
| Disordered | 1 |  | |  |
